# Potential risk factors and prevalence of *Helicobacter pylori* infection among adult patients with dyspepsia symptoms in Cameroon

**DOI:** 10.1186/s12879-018-3146-1

**Published:** 2018-06-15

**Authors:** Laure Brigitte Kouitcheu Mabeku, Michelle Larissa Noundjeu Ngamga, Hubert Leundji

**Affiliations:** 1 Department of Biochemistry, Faculty of Science, Microbiology and Pharmacology Laboratory, University of Dschang, P. O. Box 67, Dschang, Cameroon; 2 Gastroenterology Department, Laquintinie Hospital of Douala, P. O. Box 4035, Douala, Cameroon

**Keywords:** Seroprevalence, *Helicobacter pylori*, Risk factors, Gastrodudenal diseases, Cameroon

## Abstract

**Background:**

*Helicobacter pylori* is a Gram negative bacterium that colonizes the stomach of approximately two-thirds of the human population and it is involved in the pathogenesis of gastroduodenal diseases. This study sought to determine potential risk factors associated with seroprevalence of *H. pylori* among dyspepsia patients in Cameroon for a better management of the disease.

**Methods:**

The study was carried out from August to December 2014 at Laquintinie Hospital and District Hospital of Bonassama in Douala metropolis. 205 patients (127 women and 78 men; mean age, 53.79 ± 11.11 years; range, 35–75 years) were enrolled. Each subject gave a written consent. The study was approved by the local Ethical Committee of Medical Sciences. A structured questionnaire was used to collect information on sociodemographic parameters and predisposing risk factors for *Helicobacter pylori* infection. For each patient, body mass index (BMI) and direct inquiry about dyspeptic symptoms were done. Blood samples were tested for *H. pylori* antibodies, and ABO/Rhesus blood group antigen typing was performed.

**Results:**

The overall prevalence was 64.39%. All patients with upper abdominal pains and frequent burping were *H. pylori* seropositive. We found that infection takes place early in childhood and adolescence, and reaches its peak at adulthood at 35 to 44 years. Sixty-two percent of women over 68 of men were infected. 80.39% of patients with family history of gastric cancer were seropositive, while 19.60% were seronegative (*p* = 0.001). Prevalence of 79.09 and 47.4% was recorded respectively for subjects with low, middle and high income levels (p = 0.001). *H. pylori* infection rate was 60.48% in blood group O patients compared with 70.37% in other blood groups (*p* = 0.203). 73% of infected subjects over 59% of uninfected ones currently take NSAIDs (*p* = 0.0509). Overcrowded households have a higher rate of 65.32% seropositivity in contrast with a lower rate of 33.33% from norm household (*p* = 0.197). 69.29% of obese and overweight patients versus 58.24% of subjects with normal weight were seropositive (*P* = 0.215).

**Conclusion:**

The results of this study demonstrate that low income, family history of gastric cancer, clinical symptoms of nausea/vomiting and flatulence/bloating were risk factors of *H. pylori* infection in this population.

## Background

*Helicobacter pylori (H. pylori)* is a Gram-negative rod bacterium which lives in the human gastric milieu. *Helicobacter pylori* colonize the stomach and provoke a local inflammation in almost all host, a continuous process increases the risk of developing atrophic gastritis, intestinal metaplasia, and noncardia gastric adenocarcinoma [1–3]. The fourth most common cancer is gastric cancer caused by *Helicobacter pylori* and it is the second cause of mortality due to cancer in the world [4]. About 50% of the world population are infected by *Helicobacter pylori* and this rate of infection is higher in underdeveloped countries than in developed countries [5]. The report of the infection rate are as follow, 15.5% for developed countries and 93.6% for underdeveloped countries [6–8]. *H. pylori* is contagious, although the exact route of transmission is not known [9]**.** A number of authors have emphasized the role of factors such as age, socio-economic status, poor hygiene/deficient sanitation, density/crowded living conditions, smoking, use of a nonsteroidal anti-inflammatory drug (NSAID), blood group O, high body mass index and family history of gastric disease in the acquisition and transmission of *H. pylori* [10–14]**.**

Limited information is available on the prevalence of this pathogen among apparently healthy and symptomatic children in two regions of Cameroon: prevalence of *H. pylori* among apparently healthy children in the Buea and Limbe health districts of Cameroon [15], prevalence of *H pylori* among children and adolescents from the age range of 6 to 18 years old with peptic ulcer disease in Yaounde [16]. But these studies were practiced on children of age less than 18. The absence of these data in our adult population has hinder the better understanding of the harmful effect of the disease in our society. Also, it slow down the planning for resources which can enable us to best fight against *Helicobacter pylori* associated diseases which are frequent in our adulthood. Moreover, the roles of potential risk factors associated with the infection have not been elucidated in those studies. As a result, guidelines and other adequate information on the prevention and control strategies of *Helicobacter pylori* infection are lacking.

Taking into account this information, studies have been initiated by us to elucidate this question in order to bridge the gap in knowledge about the prevalence of infection among adults with dyspepsia symptoms in the littoral region of Cameroon and a thorough knowledge of risk factors such as socio-economic status, poor hygiene/deficient sanitation, density/crowded living conditions, smoking, use of a nonsteroidal anti-inflammatory drug (NSAID), blood group O, body mass index and family history of gastric disease that predispose to infection among this population in Cameroon. This region was chosen because it is the most popular and economic region of the country with high promiscuity, poor sanitation and household hygiene; factors that favor the spread of *H. pylori*.

## Methods

### Study area

The study was conducted in Douala metropolis, the largest city of Cameroon and the capital of the Littoral Region. Douala is located on the banks of the Wouri River in an area covering about 210 km^2^ divided into seven districts and has more than 120 neighbourhoods [17]. Douala typically features warm and humid conditions with an average annual temperature of 27.0 °C, an average humidity of 83% and an average annual rainfull of 3600 mm [18, 19]. The city and its surrounding area has an estimated population that surpasses 3,000,000 inhabitants. It is the commercial and economic capital of Cameroon and the entire CEMAC region. It has modest oil resource in the world and very good agricultural viability which attract people from many other parts of the country. People from other countries in the region and expatriates have also permanently settled in the city due to its highly developed infrastructures and peaceful environment for successful business. Even though Douala is the economic center of Cameroon, a large percentage of its inhabitants live below the poverty line due to its steadily increasing population. Recent data shows that about 30 % of the population lives in poverty while the aforementioned percentage is doubled for rural zones [17].

### Selection of subjects

The study was carried out within the Littoral Region using two public hospitals in Douala metropolis, Laquintinie Hospital and District Hospital of Bonassama. From August to December 2014, all patients aged 35 years or older with the symptoms of dyspepsia or other symptoms referable to the proximal alimentary tract, attended Gastroenterology Department at the selected health center were referred for serologic diagnosis of *H. pylori* infection. We employed a consecutive sampling for data collection, requesting consent from all volunteer patients in the selected health facilities who fulfilled the eligibility criteria for the study during the study period. Pregnant and breastfeeding women were excluded from the study. The number of patients enrolled was not based on a pre-selected statistical power.

### Variables

The following information were requested from the subjects in a structured questionnaire; age, sex, native region, socioeconomic class or income level [low income (≤ 2500 $/month) taking as a reference, Middle income (2500–8500 $/month) and High income (≥ 8500 $/month)], living conditions during childhood (childhood in rural or in urban zone), household population or Number of members of family that sleep in a room (household population ≤ 4 taking as a reference, household population between 5 and 7, and household population ≥ 8), smoking, Non-steroidal anti-inflammatory drugs (NSAIDs) consumption and relatives of gastric cancer. For all participants, direct inquiry about dyspeptic symptoms: upper abdominal pains (epigastric pains, burning), abdominal discomfort (flatulence, bloating), nausea/vomiting and frequent burping was done by a resident gastro-enterologist and the clinical characteristics were recorded. Height and weight were measured for each participant, and BMI was calculated as weight/height^2^ (kg/m^2^). Participants were classified relative to BMI value in 3 groups; normal (< 25 kg/m^2^), overweight (25 to 30 kg/m^2^) and obese (> 30 kg/m^2^). The participants with normal BMI were taken as reference group for statistical analysis.

### Collection of sample/analysis

Three milliliters (3 ml) of venous blood was drawn from each patient, then 2 ml of it transferred into a clean test tube and allowed to clot naturally by standing the sample at room temperature for 10 mn. Clear serum sample was obtained by spinning the tubes at 3000 rpm for 5 mn and used for the detection of antibodies against *H. pylori* using One Step *H. pylori* test device (*DiaSpot H. pylori,* Indonesia). The remaining 1 ml of the blood was emptied into disposable tubes, and used for determination of ABO blood group antigens by a standard hemagglutination test. Participants were classified relative to ABO blood group antigens in 4 groups (Group A, Group B, Group AB, and Group O was taken as reference group for statistical analysis) and in positive or negative according to the rhesus status.

For each sera collected, 3 drops were used to detect *Helicobacter pylori* antibodies according to the manufacturer’s instruction. DiaSpot^R^ *H. pylori* One Step Test Device is a rapid chromatographic immunoassay for the qualitative detection of *H. pylori* antibodies in serum or plasma. It is a test that utilizes a combination of *H. pylori* antigen coated particles and anti-human IgG to qualitatively and selectively detect *H. pylori* antibodies in serum or plasma. Anti-human IgG is immobilized in the test line region. The sample is added to a specific area on the test device, it reacts with *H. pylori* antigen coated particles in the test. This mixture migrates chromatographically along the length of the test and interacts with the immobilized anti-human IgG. Appearance of a single red line indicates a negative result while double lines indicate positive sample. This test has sensitivity > 95.9% and specificity about 75.9% with overall accuracy of 85.2% as compared with culture/histology of endoscopic specimens for *H. pylori*.

The ABO blood group was determined for each patient by the conventional hemagglutination test using the anti-A, anti-B and anti-D sera. The ABO blood grouping procedure is based on the principle of agglutination or clumping as the patient’s blood is reacted with anti-A, anti-B and anti-Rh antibodies separately.

### Statistical analysis

The data generated were coded, entered, validated and analyzed using SPSS 23. We tested for association in categorical variables using the chi-squared test, reporting corresponding *p*-values. In case of small numbers in a given group (< 5), the Fischer’s exact test was used, and the corresponding p-value reported. For the purpose of hypothesis testing, a control group constituted by uninfected participants was used to compare the strength of the association between reported risk factors and infection with *H. pylori*.

The odds ratio and the corresponding 95% confidence intervals (95% CI) were used to summarize the strength of association between specific binary exposure and outcome variables. The level of statistical significance for the study was set at *p* < 0.05. A multivariate logistic regression analysis was done for potential risk factors with *p* value less than 0.05 or near 0.05 to confirm the variable as risk factors of *H. pylori* infection. The outcome measure was the detection of the presence of *H. pylori* antibodies in the serum of patients with gastric-related morbidities, and their association with known risk factors for infection.

## Results

### Sociodemographic and clinical characteristics of the study population

A total of 205 patients were enrolled. Their mean age was 53.79 ± 11.11 years (range 35–75 years), and the age group from 55 to 64 years the most represented (35.6%; 75/205). With 78 male and 127 female subjects, the overall male/female ratio was 1:1.7. All the 10 regions of Cameroon were represented in our sample population; 60% from the West Region, 25.36% from the Littoral, 12.20% from the Center, East and South, 1% from the South and North West, 1% from the Far North, North and Adamawa. 0.5% of the patients were from others countries. Fifty four percent (53.65%; 110/205) of the subjects were unemployed (low income or poorly skilled class) followed by 26.34% (54/205) from middle class with 2500–8500 $/month and 20% (41/205) from elite class with relatively high income (≥ 8500 $/month). Half (102/205) of our sample were living in rural zone during childhood. A similar distribution was recorded as far as family history of gastric cancer was concerned. A household of three to four in a Cameroonian standardized room is considered as the norm; households that are crowded have 5–7 people, and the overcrowded, up to 8 people. In our sample, 70% (143/205) of households were overcrowded with 8 to 15 people, followed by crowded ones with 27.31% (56/205). Few number of our population currently smoke and take NSAIDs with a frequency of 17.05% (35/205) and 38.04% (78/205) respectively. The distribution of the ABO blood groups among patients was 60.5% for O group, followed by B (18.54%), A (17.56%) and AB (3.41%), while 203 (99.02%) of patients were rhesus positive and 2 (0.975%) rhesus negative. Among the 205 participants, 4.88% (10) and 50.73% (104) were respectively obesed and overweight, while 44.4% (91) were with normal weight (Table 1).

**Table 1:** Characteristics of the study population

Variable	Number (%)
Age (years) Mean (53.79 ± 11.11) years; Range: 35 to 75 years	
35–44	47 (22.92)
45–54	49 (23.90)
55–64	75 (36.58)
65–74	29 (14.14)
75–84	5 (2.44)
Sex	
Female	127 (61.95)
Male	78 (38.04)
Origin	
West	123 (60)
Littoral	52 (25.36)
Center, East and South	25 (12.20)
South and North West	2 (0.1)
Far Nord, North and Adamawa	2 (0.1)
Others countries	1 (0.5)
Socio-economic class/Income level ($/month)	
Unemployed or low class (≤ 2500)	110 (53.65)
Middle class (2500–8500)	54 (26.34)
Elite (≥ 8500)	41 (20)
Household population (family size)	
≤ 4	6 (2.92)
5–7	56 (27.31)
≥ 8	143 (69.75)
Smoking	
Yes	35 (17.07)
No	170 (82.92)
Living conditions during childhood	
Rural zone	102 (49.75)
Town zone	103 (50.24)
NSAIDs) consumption	
Yes	78 (38.04)
No	127 (61.95)
Family history of gastric cancer	
Yes	102 (49.75)
No	103 (50.24)
ABO blood group	
Group O	124 (60.48)
Group A	36 (17.56)
Group B	38 (18.54)
Group AB	7 (3.41)
Rhesus (+)	203 (99.02)
Rhesus (−)	2 (0.975)
Body mass index; Mean (25.0924 ± 0.8411) kg/m^2^; Range: 18.59 to 40.63 kg/m^2^	
Normal	91 (44.4)
Overweight	104 (50.73)
Obese	10 (4.88)

*H.pylori: Helicobacter pylori*; SD: standard deviation

Direct inquiry about dyspeptic symptoms was done on all the 205 enrolled patients and all of them presented at least two of the clinical characteristics. On the clinical signs, epigastric pain/burning and frequent burping were the most common (100%; 205/205), followed by abdominal discomfort (104/205) and nausea/vomiting (49/205) (Table 2).

**Table 2:** Relationship between clinical signs of the study population and *H. pylori* status

Clinical signs	Number (%)	*H. pylori* (+) (N = 132)	*H. pylori* (−) (*N* = 73)	OR (95% CI) *P* value
Epigastric pains/Burning				/
Yes	205 (100)	132 (100)	73 (100%)	
No	0 (0)	0 (0.00)	0 (0.00)	
Frequent burping				/
Yes	205 (100)	132 (100)	73 (100%)	
No	0 (0)	0 (0.00)	0 (0.00)	
Flatulence/ bloating				13.44 (6.397–28.24) ˂ 0. 0001
Yes	104 (51)	93 (70.45%)	11 (15.07%)	
No	101 (49)	39 (29.54%)	62 (85%)	
Nausea/Vomiting				8.922 (3.059–26.03) ˂ 0.0001
Yes	49 (24)	45 (34.1%)	4 (5.48%)	
No	156 (76)	87 (65.90%)	69 (94.52%)	
*H. pylori* status				
Positive	132 (64.39)			
Negative	73 (35.61)			

*H. pylori*: *Helicobacter pylori*, (95% CI): 95% confidence intervals, /: not determined

### Seroprevalence of *H. pylori* infection of the study population

205 patients with various gastroduodenal symptoms were tested for *H. pylori* infection using a rapid chromatographic immunoassay for the qualitative detection of antibodies against *H. pylori.* Of these patients, 132 were *H. pylori* positive, giving an overall prevalence of 64.39% (Table 2).

As regards the clinical signs, all *H. pylori* (100%; 132/132) infected and uninfected patients presented epigastric pain/burning and frequent burping as dyspeptic signs, thus these two symptoms cannot be used to predict the etiology of epigastralgia as *H. pylori*-related in our sample population. However, when considering the other clinical signs evaluated, we noticed that infected patients were prone to develop Flatulence/ bloating (70.45 versus 15.07%) and Nausea/Vomiting (34.1 versus 5.48%) than uninfected ones (Table 2). Such variations within the distribution of Flatulence/ bloating and Nausea/Vomiting among infected and uninfected patients may imply that these clinical signs are highly suggestive of *H. pylori* infection in the study population.

The strength of the association between socio-demographic parameters evaluated and infection with *H. pylori,* shows different rates of infection relative to the age group and sex of the participant, but this difference was not statistically significant (Table 3).

**Table 3:** Relationship between socio-demographic characteristics and *H. pylori* status of the study population

Variable	*H. pylori* (+) (N = 132) (%)	*H. pylori* (*−*) (N = 73) (%)	X^2^; P value
Age (years)			4.057; 0.398
35–44	35 (74.46)	12 (25.53)	
45–54	28 (57.14)	21 (42.85)	
55–64	46 (61.33)	29 (38.66)	
65–74	19 (65.51)	10 (34.48)	
75–84	4 (80)	1 (20)	
Sex			0.6953; 0.404
Female	79 (62.20)	48 (37.79)	
Male	53 (67.94)	25 (32.05)	
Origin			
West	77 (62.60)	46 (37.39)	
Littoral	35 (67.30)	17 (32.69)	
Center, East and South	15 (60)	10 (40)	
South and North West	2 (100)	0 (0)	
Far Nord, North and Adamawa	2 (100)	0 (0)	
Others countries	1 (100)	0 (0)	

The highest prevalence was obtained in the age group of 75–84 (80%), but the number of patients aged ≥75 years studied, has been much smaller than those younger than 75 years. So, we found that frequency of seropositivity for *H. pylori* seems to be at the same level among participants aged over 35 years. Comparing seropositivity in males and females, our study confirms the fact that more males (67.94%) are being seropositive than females (62.20%). But the difference was not significant (*P* = 0.4044).

The association between *H. pylori* infection and the potential risk factors are summarized on Table 4. Contrary to smoking and living conditions during childhood which did not affect *H. pylori* status, the other risk factors evaluated have a positive effect on the frequency of *H. pylori* infection, with a higher risk of being seropositive among exposed patients than unexposed ones, but the strength of the association was only statistically significant with the income level and family history of gastric cancer (Table 4).

**Table 4:** Potential risks factor and *H pylori* status of the study population

Variable	Number	*H. pylori* (+) (N = 132) (%)	*H. pylori* (*−*) (N = 73) (%)	OR (95% CI)	*P* value
Low income (≤ 2500 $/month)				3.590 (2.063–6.765)	0.001
Yes	110	87 (79.09)	23 (20.90)		
No	95	45 (47.4)	50 (52.6)		
Household population ˃ 4 members				3.528 (0.631–12.327)	0.197
Yes	199	130 (65.32)	69 (34.67)		
No	6	2 (33.33)	4 (66.66)		
Smoking				0.9231 (0.4341–1.963)	0.8481
Yes	35	22 (62.85%)	13 (37.14%)		
No	170	110 (64.70%)	60 (35.29%)		
Living conditions during childhood				0.9439 (0.5328–1.672)	0.8846
Rural zone	102	65 (63.72)	37 (36.27)		
Urban zone	103	67 (65.04)	36 (34.95)		
NSAIDs consumption				1.882 (1.020–3.473)	0.0509
Yes	78	57 (73.07%)	21 (26.92%)		
No	127	75 (59.05%)	52 (40.94)		
O blood group				0.681 (0.373–1.261)	0.203
Yes	124	75 (60.48%)	49 (34.50%)		
No	81	57 (70.37)	24 (29.62)		
Rhesus status				0.626 (0.554–0.691)	0.531
Rhesus (+)	203	130 (64.03)	73 (35.96)		
Rhesus (−)	2	2 (100.00)	0 (0.00)		
Family history of gastric cancer				2.758 (1.563–5.361)	0.001
Yes	102	82 (80.39)	20 (19.60)		
No	103	50 (48.54%)	53 (51.45%)		
Body mass index ≥25 kg/m^2^				1.434 (0.824–2.548)	0.215
Yes	114	79 (69.29)	35 (30.70)		
No	91	53 (58.24)	38 (41.75)		

Our data showed that, among the 102 patients with family history of gastric cancer, 82 (80.39%) were seropositive, while 20 (19.60%) were seronegative. This difference was significant (*P* = 0.001).

Similarly, socioeconomic status of participants seems to play an important role on the frequency of infection in this study. Prevalence of 79.09% in unemployed subjects with low income was recorded, in contrast with lower rate of 47.40% from subjects with middle and high income. This difference was significant (*p* = 0.001).

*H. pylori* infection was also associated to NSAIDs consumption, with a higher risk of seropositivity amongst NSAIDs users (73.07%: 57/78) relative to non-users (59.05%: 75/127); the strength was not significant (*P* = 0.0509).

A multivariate logistic regression analysis was done for the variables; income level, NSAIDs consumption and family history of gastric cancer. The results are summarized on Table 5. From this table, we noticed that among these three categorical variables, income level (*p* = 0.001) and family history of gastric cancer (*p* = 0.002) were still associated to *H pylori* infection with a statistically significant strength.

**Table 5:** Multivariate logistic regression analysis of risk factors of *H pylori* infection

Variable	*H. pylori* (+) (%)	*H. pylori* (*−*) (%)	OR (95% CI)	*P* value
Low income (≤ 2500)				
Yes	87 (79.09)	23 (20.90)	1,794,969,562 (20.893 to 21.724)	0.001
No	45 (47.4)	50 (52.6)		
NSAIDs consumption				
Yes	57 (73.07%)	21 (26.92%)	0.509 (−2.612 to 0.328)	0.230
No	75 (59.05%)	52 (40.94)		
Family history of gastric cancer				
Yes	82 (80.39)	20 (19.60)	0.000 (−20.510 to −17.871)	0.002
No	50 (48.54%)	53 (51.45%)		
Household population > 4				
Yes	130 (65.32)	69 (34.67)	0.000 0.000 to	0.999
No	2 (33.33)	4 (66.66)		
Nausea/Vomiting				
Yes	45 (91.84%)	4 (8.16%)	5.41 2.05 to 14.24	0.001
No	87 (55.77%)	69 (44.23%)		
Flatulence/Bloating				
Yes	93 (89.42%)	11 (10.58%)	7.705 3.77 to 15.74	0.0001
No	39 (38.61%)	62 (61.39%)		

As ABO blood group status is concerned, our data showed that, among the 124 participants with type O blood group, 75 (60.48%) were seropositive, while 49 (34.50%) were seronegative (Table 4). *H. pylori* infection rate was 60.48% in blood group O patients compared with 70.37% in other blood groups (Table 4). However, this difference was not significant (*P* = 0.203). The *H. pylori* infection rate in other blood groups were 72.22% (26/36) in group A, 68.42% (26/38) in group B, and 71.42% (5/7) in group AB, which were slightly higher than blood group O, but without statistical significance (Table 1).

Regarding rhesus (Rh) status, 64.03% Rh (+) and 100% Rh (−) patients were positive for *H. pylori.* Although Rh (−) patients in our sample seemed to have high seropositivity, this blood group was not representative because it was not common in the study population.

We also noticed that overcrowding increases the chance of seropositivity among the households population, although this difference was not significant (*P* = 0.197). Households with > 4 members have a higher rate of 65.32% seropositivity in contrast with lower rate of 33.33% from household with ≤4 members (Table 4).

Our data demonstrated that obesity and/or a high BMI is associated with an increased incidence of *H. pylori* infection. A seropositive rate of 69.29% (79/114) was recorded among obesed and overweight patients, in contrast with a lower rate of 58.24% from subjects with normal weight. However, this difference was not significant (*P* = 0.215).

## Discussion

The diagnosis of gastric infection with *H. pylori* usually involves upper endoscopy. However, serological methods have reached sufficient accuracy to be used as screening tests before endoscopy, or for sero-epidemiological surveys as individuals infected with *H. pylori* develop antibodies which correlate strongly with histologically confirmed *H. pylori* infection [20–24].

In our study we evaluated the seroprevalence of *Helicobacter pylori* infection among patients aged 35 years and older with dyspepsia symptoms in Douala metropolis, and we found a seroprevalence of 64.39% which is lower than the prevalence of 92.2 and 79.3% reported respectively by Ndip et al. (2004) [15] in the North-west region, and Andoulo et al. (2015) in the Center region of Cameroon [16], both among children less than 18 years old. The age range difference in the studied population between the present and these previous studies, may explain such observation. In fact, in our sampling processes, we included only patients aged 35 years or older, who are thought to better observe principles of cleanliness compared to younger ones. Thus, the incidence rate among patients aged ≥35 years would be more or less lower than that in the younger population. Moreover, the differences in these value can also be due to the study area, the socioeconomic conditions in the study area and the type of controls used, since the prevalence of *Helicobacter pylori* is known to represent the improvements in socioeconomic conditions and sanitary standards throughout the generations. In Russian for instance, within a period of ten years (from 1995 to 2005), it was observed that the prevalence of *Helicobacter pylori* infection has reduced remarkably due to better standards of living [25]. A similar situation was observed in China due to an increase in economic growth, and improvement in environmental and hygienic conditions [26].

The seroprevalence we found correlates with the results of some studies done in other developing countries within and out of Africa. A seroprevalence of 62.4% in the Democratic Republic of Congo [27], 50.6% in the North of South Africa (Venda) [28], about 58% in Guatemala [29], 68% in Turkey [30], 61% in Saudi Arabia [31], 62% in Kuwait [32], 66.7 to 85% in some regions of Iran [33, 34], and 61.7% in Brazil [35] was observed among the examined individuals.

However, our prevalence was found to be low as compared to those of other studies. For instance, an infection rate of 93% was observed in Ethiopian on patients with peptic ulcer disease [36]. Also, an infection rate of 97 and 94.5% were reported in Ghana [37] and in Mozambique [38] respectively. A prevalence of *H. pylori* antibodies in an interval of 67 to 84% was found among children in South Africa (Bloemfontein) by Pelsar et al. (1997) [39]. Asymptomatic individuals in Eastern Cape Province were studied and 86.8% of *Helicobacter pylori* antigenemia was observed in their stools [12]. Ghose et al. (2005) [40] in Venezuela also found a high prevalence of 95.3% in their study population. The differences between the prevalence of the present study and the ones mentioned above may be as a result of different detection method and geographical area which could reflect the environmental and personal hygiene.

The relationship between clinical signs of the study population and *H. pylori* status shows that, unlike Upper abdominal pains and frequents Burping, Flatulence/ bloating and Nausea/vomiting are significantly suggestive of *H. pylori* infection in the study population (Table 5). However, a meta-analysis reported that children with upper abdominal pain or epigastric pain were two to three folds more infected to *H. pylori* than children without these symptoms [41]. The age range difference in the studied population between the present study and this meta-analysis may explain such observation. In fact, in our sampling processes, we included patients aged 35 years or older and they were considered to be exposed to other etiologic agents of epigastralgia such as NSAIDs abuse than younger population.

Our data on gender agrees with some studies indicate that the males were associated with a higher risk of acquiring *H. pylori* infection than the females [15], because the males are naturally more active and less hygienic than females, since the prevalence of *H. pylori* infection and sanitation condition are inversely related. However, some documented studies disagree with this idea, but the reasons is still not known [42, 43].

Most infection cause by *Helicobacter pylori* are highly related to poor living conditions in childhood [44] and in the absence of antibiotics therapy, *Helicobacter pylori* may persist for life in the host [45]. According to the age group in our study, the newly acquired *Helicobacter pylori* infection had occurred during childhood and early adolescence, in adulthood (35 to 44 years), it increases and reaches a peak and remains almost the same as you grow older. The present result was in accordance with many other authors’ research output. In a rural village of Linqu Country, Shandong Province, China a study on 98 children was made, 70% of those aged between 5 and 6 years were found to be infected with the organism and a similar rate was observed on adult in the same area, so it was concluded that most infection occur at childhood [46].

As regards socioeconomic and family history of gastric cancer status of participants, our data shows that those two factors were significant risk factors for *H. pylori* infection even after a multivariate logistic regression analysis. We noticed an increase of seropositivity rate from higher class to low or poorly skilled class, suggesting a dreadful trend of *H. pylori* infectivity due to poor socio-economic status of participants. This is similar to reports from other parts of the globe, indicating association of low socio-economic factors with prevalence of *H. pylori* infection in Asia [47], South America [48] and Africa [49]. In fact, poverty enhanced level of transmission due to malnutrition, poor hygiene and unaffordable heath care. Similarly, we also noticed that participants with family history of gastric cancer had a greater tendency towards *H. pylori* infection. Other studies showed that family history of peptic disease and gastric cancer was significantly associated with *H. pylori* infection [50, 51].

Our finding shows that, the prevalence tended to be higher if there were more family members in a household, although this difference was not significant (Table 5). This confirms the works of several authors that overcrowding facilitates transmission of *H. pylori* [52, 53]. Some other studies have reported household crowding and bed sharing among different people as strong and independent risk factors for *H. pylori* [54, 55]. This is because household members share eating utensils, live in poorly ventilated rooms and are more likely prone to poor hygiene.

Gastroduodenal diseases are mainly caused by *Helicobacter pylori* infection and the use of a nonsteroidal anti-inflammatory drug (NSAID). A meta-analysis [56] that included 463 studies evaluating the association between *H. pylori* infection and NSAID used, revealed a higher rate of peptic ulcer disease among NSAID user when *Helicobacter pylori* infection was present (41.7%) as compared to users who were *H pylori*-negative (25.9%) [56]. Peptic ulcer disease was 3.5 times more present in NSAID users who were *H pylori*-positive [57]. In accordance with what was said previously, seropositivity increases with the use of NSAID among our study population. The complementary effect between the two causative factors which lead to the development of gastroduodenal diseases may take place through the production of oxygen reactive species (ROS). In fact, oxygen reactive species favors the development of pathogenesis in acute experimental gastric lesions induced by NSAIDs [57]. Moreover, experimental results strongly support the implication of ROS in the pathogenesis of the *H. pylori*-induced chronic gastritis [58].

Our finding showed that blood group O was not associated with higher *H. pylori* infection rate. *H. pylori* infection rate was 60.48% in blood group O patients compared with 70.37% in other blood groups (*p* = 0.203), (Table 4). The *H. pylori* infection rate in other blood groups were 72.22% (26/36) in group A, 68.42% (26/38) in group B, and 71.42% (5/7) in group AB, which were slightly higher than in blood group O, but without statistical significance. The lower rates of other blood groups in the *H. pylori* infected patients (*n* = 132) simply reflect the lower prevalence of these blood group types in the study population (Table 1). Previous studies suggested that blood group O do not represent a risk factor for *Helicobacter pylori* infection [30, 59] which agree with our result but disagree with other studies who tie on the fact that individual of blood group O have a higher incidence of developing duodenal and gastric ulcers [43, 60–63]. This increase in the development of the disease in the blood group O individuals may be due to a great colonization of their epithelial cells and higher inflammatory responses to *H. pylori* [2].

According to Cohen et al. [64] study, higher BMI levels are common in *Helicobacter pylori* infected adults despite the fact that the subject may be asymptomatic or not and further suggested that eradication of *Helicobacter pylori* may lead to loss in weight. Similarly, our study has demonstrated that the probability to *Helicobacter pylori* infection increase with high BMI level. Some data show that *Helicobacter pylori* infection can affect the production of leptin and ghrelin and subsequently their plasma level (increasing plasma ghrelin and decreasing leptin levels) [65–69]. The fluctuation in the plasma level of this hormone could promote obesity. In fact, Ghrelin decreases energy loss and promotes weight gain [70], whereas leptin reduces food intake and increases energy loss [71]. In contrast, other studies show that there is no relationship between *H. pylori* seropositivity or CagA antibody status and high BMI [72, 73] and not even an inverse relationship between morbid obesity and *H. pylori* seropositivity [74].

## Conclusion

Our study shows that the prevalence of *H. pylori* among our sample population is high and that low income, family history of gastric cancer, clinical symptoms of nausea/vomiting and flatulence/bloating were risk factors of *H. pylori* infection in this population.

## References

[1] Akbar DH, El Tahawy AT (2005). *Helicobacter pylori* infection at a university hospital in Saudi Arabia, prevalence, comparison of diagnostic modalities and endoscopic findings. Indian journal of pathology and. Microbiology.

[2] Alkout AM, Blackwell CC, Weir DM (2000). Increased inflammatory responses of persons of blood group O to *Helicobacter pylori*. J Infect Dis.

[3] Alkout AM, Blackwell CC, Weir DM, Poxton IR, Elton RA, Luman W, Palmer K (1997). Isolation of a cell surface component of *Helicobacter pylori* that binds H type 2, Lewis a and Lewis b antigens. Gastroenterology.

[4] Atherton JC, Blaser MJ (2009). Coadaptation of *Helicobacter pylori* and humans: ancient history, modern implications. J Clin Investig.

[5] Fock KM, Ang TL (2010). Epidemiology of *Helicobacter pylori* infection and gastric cancer in Asia. J Gastroenterol Hepatol.

[6] Eusebi LH, Zagari RM, Bazzoli F (2014). 2014. Epidemiology of *Helicobacter pylori* infection. Helicobacter.

[7] Mentis A, Lehours P, Mégraud F (2015). Epidemiology and diagnosis of *Helicobacter pylori* infection. Helicobacter.

[8] Tonkic A, Tonkic M, Lehours P, Mégraud F. Epidemiology and diagnosis of *Helicobacter pylori* infection. Helicobacter. 2012:17:1–8. 10.1111/j.1523-5378.2012.00975.x 22958148

[9] Megraud F (1995). Transmission of *Helicobacter pylori*: Faecal-oral versus oral-oral route. Alimentary. Pharmacol Ther.

[10] Segal I, Ally R, Mitchell H (2001). *Helicobacter pylori*: an African perspective. Q J Med.

[11] Chong VH, Lim KC, Rajendran N (2009). Prevalence of active *Helicobacter pylori* infection among patients referred for endoscopy in Brunei Darussalam. Singap Med J.

[12] Dube C, Nkosi TC, Clarke AM, Mkwetshana N, Green E, Ndip RN (2009). *Helicobacter pylori* in an asymptomatic population of eastern Cape Province,South Africa: public health implication. Rev Environ Health.

[13] Ogihara A, Kikuchi S, Hasegawa A, Kurosawa M, Miki K, Kaneko E, Mizukoshi H (2000). Relationship between *Helicobacter pylori* infection, smoking and drinking habits. J Gastroenterol Hepatol.

[14] Brown LM (2000). *Helicobacter pylori:* epidemiology and routes of transmission. Epidemiol Rev.

[15] Ndip RN, Malange AE, Akoachere JFT, MacKay WG, Titanji VPK, Weaver LT (2004). *Helicobacter pylori* antigens in the faeces of asymptomatic children in the Buea and Limbe health districts of Cameroon: a pilot study. Trop Med Int Health.

[16] Ankouane Andoulo F, Ngatcha G, Tagni-Sartre M, Biwolé Sida M, Ndjitoyap Ndam EC. *Helicobacter pylori* infection and peptic ulcer disease in children and adolescents from the age range of 6 to 18 years old in Yaounde (Cameroon). 2015.

[17] Zelaza PT. Douala, Cameroon in Encyclopedia in Twentieth Century African History, eds. Routledge: Dickson Eyoh; 2002. p. 151.

[18] World Weather Information Service-Douala. World Meteorological Organization. Retrieved 13 2016.

[19] Klamatefef von Douala (Duala), Obsevateriun / Kamerun, Baseline climate means (1961-1990) from stations over the world (in German). Deutscher wetterdienst. Retrieved 13 june 2016.

[20] Philipp ML, Angelika M, Konstanze V (2004). Comparison of different criteria for interpretation of IgG immunoblotting results for diagnosis of *H. pylori* infection. Infec Immun.

[21] Loffeld RJLF (1993). Usefulness of several commercial ELISA for detection of *H. pylori* in clinical medicine. Euro J Gastroen Hepa.

[22] Cutler AF (1995). Accuracy of invasive and non invasive tests to diagnose *H. pylori*. Gastroenterology.

[23] Ansorg R, Von Recklinghausen G, Pomarius R (1991). Evaluation of techniques for isolation, subcultivation and preservation of *H. pylori*. J Clin Micro..

[24] Pronovast AP, Rose SL, Pawlak J (1994). Evaluation of a new immunodiagnostic assay for *H. pylori* antibody detection: correlation with histopathological and microbiological results. J Clin Micro.

[25] Tkachenko MA, Zhannat NZ, Erman LV, Blashenkova EL, Isachenko SV, Isachenko OB, Graham DY, Malaty HM (2007). Dramatic changes in the prevalence of *Helicobacter pylori* infection during childhood: a 10-year follow-up study in Russia. J Pediatr Gastroenterol Nutr.

[26] Nagy P, Johansson S, Molloy-Bland M (2016). Systematic review of time trends in theprevalence of *Helicobacter pylori* infection in China and the USA. Gut Pathogens.

[27] Longo-Mbenza B, Nsenga JN, Ngoma VD (2007). Prevention of metabolic syndrome insulin resistance and atherosclerotic in diseases in Africans infected by *Helicobacter pylo*ri infection and treated with antibiotics. Inter J Cardiol.

[28] Samie A, Obi CL, Barrett LJ, Powell SM, Guerrant RL (2007). Prevalence of *Campylobacter* species, *Helicobacter pylori* and *Arcobacter* species in stool samples from the Venda region, Limpopo, South Africa: studies using molecular diagnostic methods. J Inf Secur.

[29] Dowsett AS, Archila L, Segreto AV, Gonzalez RC, Silva A, Vastola AK, Bartizek DR, Kowolik JM (1999). *Helicobacter pylori* infection in indigenous families of central America: Serostatus and oral and fingernail carriage. J Clin Microbiol.

[30] Seyda T, Derya C, Füsun A, Meliha K (2007). The relationship of *Helicobacter pylori* positivity with age, sex, and ABO/rhesus blood groups in patients with gastrointestinal complaints in Turkey. Helicobacter.

[31] Khan MA, Ghazi HO (2007). *Helicobacter pylori* infection in asymptomatic subjects in Makkah, Saudi Arabia. J Pak Med Assoc.

[32] Alazmi WM, Siddique I, Alateeqi N, Al-Nakib B (2010). Prevalence of *Helicobacter pylori* infection among new outpatients with dyspepsia in Kuwait. BMC Gastroenterol.

[33] Farshad SH, Japoni A, Alborzi A-V, Zarenezhad M, Ranjbar R (2010). Changing prevalence of *Helicobacter pylori* in south of Iran Iranian. J. Clin Infect Dis.

[34] Massarrat S, Saberi-Firoozi M, Soleimani A, Himmelmann GW, Hitzges M, Keshavarz H (1995). Peptic ulcer disease, irritable bowel syndrome and constipation in two populations in Iran. Eur J Gastroenterol Hepatol.

[35] de Mattos LC, Cintra JR, Sanches FE (2002). ABO, Lewis, secretor and non-secretor phenotypes in patients infected or uninfected by the *Helicobacter pylori* bacillus. Sao Paulo Med J.

[36] Henriksen T, Nysaeter G, Madebo T, Setegn D, Brorson O, Kebede T, Berstad A (1999). Peptic ulcer disease in South Ethiopia is strongly associated with *Helicobacter pylori*. Trans Roy Soc Trop Med Hyg.

[37] Kidd M, Louw JA, Mark NI (1999). *Helicobacter pylori* in Africa: observation on an ‘enigma within an enigma. J Gastroenterol Hepatol.

[38] Carrilho C, Modcoicar P, Cunha L, Ismail M, Guisseve A, Lorenzoni C, Fernandes F, Peleteiro B, Almeida R, Figueiredo C, David L, Lunet N (2009). Prevalence of *Helicobacter pylori* infection, chronic gastritis, and intestinal metaplasia in Mozambican dyspeptic patients. Virchows Arch.

[39] Pelsar HH, Househam KC, Joubert G, van der Linde G, Kraaj P, Meinardi M (1997). Prevalence of *Helicobacter pylori* antibodies in children in Bloemfontein, South Africa. J Pediatr Gastroenterol Nutr.

[40] Ghose C, Perez-Perez GI, van Doorn LJ, Domı’nguez-Bello MG, Blaser MJ (2005). High frequency of gastric colonization with multiple *Helicobacter pylori* strains in Venezuelan subjects. J Clin Microbiol.

[41] Spee LA, Madderom MB, Pijpers M, Van Leeuwen Y, Berger MY (2010). Association between *Helicobacter pylori* and gastrointestinal symptoms in children. Pediatrics.

[42] Kanbay M, Gur G, Arslan H (2005). The relationship of ABO blood group, age, gender, smoking, and *Helicobacter pylori* infection. Dig Dis Sci.

[43] Lacy B, Semore J (2001). *Helicobacter pylori*: ulcers and more: the beginning of an era. J Nutr.

[44] Webb PM, Knight T, Greaves S, Wilson A, Newell DG, Elder J (1994). Relation between infection with *Helicobacter pylori* and living conditions in childhood: evidence for person to person transmission in early life. Br Med J.

[45] Pacifico L, Anania C, Osborn JF, Ferraro F, Chiesa C (2010). 2010. Consequences of *Helicobacter pylori* infection in children. World J Gastroenterol.

[46] Dale A, Thomas JE, Darboe MK, Coward WA, Harding M, Weaver LT (1998). Helicobacter pylori infection, gastric acid secretion, and infant growth. J. Pediatr. Gastroenterol. Nutr.

[47] Holcombe C, Tsimiri S, Eldridge J, Jones DM (1993). Prevalence of antibody to *Helicobacter pylori* in children in northern Nigeria. Trans R Soc Trop Med Hyg.

[48] Katelaris PH, Tippett GHK, Norbu P, Lowe DG, Brennan R, Farthing MJG (1992). Dyspepsia, *Helicobacter pylori* and peptic ulcer in a randomly selected population in India. Gut.

[49] Louw JA, Jaskiewicz K, Girdwood AH (1993). *Helicobacter pylori* prevalence in nonulcer dyspepsia-ethnic and socioeconomic differences. S. Afr Med J.

[50] Dore MP, Fanciulli G, Tomasi PA, Realdi G, Delitala G, Graham DY, Malaty HM (2012). Gastrointestinal symptoms and *Helicobacter pylori* infection in school- age children residing in Porto Torres, Sardinia, Italy. Helicobacter.

[51] Habib HS, Hegazi MA, Murad HA, Amir EM, Halawa TF, El-Deek BS (2014). Unique features and risk factors of *Helicobacter pylori* infection at the main children's intermediate school in Rabigh, Saudi Arabia. Indian J Gastroenterol.

[52] Ogunlesi TA, Dedeke IOF, Kuponiyi OT (2008). Socioeconomic classification of children attending specialist paediatric centres in Ogun state, Nigeria. Niger Med Pract.

[53] Brown LM, Thomas TL, Ma JL, Chang YS, You WC, Liu WD, Zhang L, Pee D, Gail MH (2002). *Helicobacter pylori* infection in rural China: demographic, lifestyle and environmental factors. Int J Epidemiol.

[54] McCallion WA, Murray LJ, Bailie AG, Dalrell AM, O‘Reilly DPJ, Bamford KB. *Helicobacter pylori* infection in children: relation with current household living conditions. Gut 1996;39:18–21. 10.1136/gut.39.1.18 PMC1383223 8881801

[55] Moayyedi P, Anthony TR, Axon AT (2002). Relation of adult lifestyle and socioeconomic factors to the prevalence of *Helicobacter pylori* infection. Int J Epidemiol.

[56] Loeb DS, Talley NJ, Ahlquist DA, Carpenter HA, Zinsmeister AR (1992). Long-term nonsteroidal anti-inflammatory drug use and gastroduodenal injury: the role of *Helicobacter pylori*. Gastroenterology.

[57] Banarjee RK (1990). Non-steroidal anti-inflammatory drugs inhibit gastric peroxidase activity. Biochim Biophys Acta.

[58] Lignell A, Surace R, Bottiger P, Borody TJ. Symptom improvement in *Helicobacter pylori*-positive non-ulcer dyspeptic patients after treatment with the carotenoid astaxanthin. In: 12th international carotenoid symposium, cairns, Australia, 18–23 1999.

[59] Sharara A, Abdul-Baki H, El Hajj L, Kreidielh N, Kfoury Baz EM (2006). Association of gasterodudenal disease phenotype with ABO blood group and *Helicobacter pylori* virulence specific serotypes. Dig Liver Dis.

[60] Mattos LC, Cintra JR, Sanches FE, Alves RC, Ruiz MA, Moreira HW (2002). ABO, Lewis, secretor and non-secretor phenotypes in patients infected or uninfected by the *Helicobacter pylori* bacillus. Sao Paulo Med J.

[61] Alkout AM, Blackwell CC, Weir DM, Poxton IR, Elton RA, Luman W, Palmer K (1997). Isolation of a cell surface component of *Helicobacter pylori* that binds H type 2, Lewis a 4. And Lewis b antigens. Gastroenterology.

[62] Dhillon BS, Shergill SS (2004). Prevalence of ABO and Rh blood groups in color vision defective Punjabi population. North Zone Ophthalmological Soc J.

[63] Martins LC, Corvelo TCO, Oti HT, Loiola RSP, Aguiar DCF, Barile KAS, Amaral RKC, Barbosa HPM, Fecury AA, de Souza JT (2006). ABH and Lewis antigen distributions in blood, saliva and gastric mucosa and H. Pylori infection in gastric ulcer patients. World J Gastroenterol.

[64] Cohen D, Muhsen K (2012). Association between *Helicobacter pylori* colonization and glycated hemoglobin levels: is this another reason to eradicate *H. pylori* in adulthood?. J Infect Dis.

[65] Roper J, Francois F, Shue PL, Mourad MS, Pei Z, Olivares de Perez AZ, Perez-Perez GI, Tseng CH, Blaser MJ (2008). Leptin and ghrelin in relation to *Helicobacter pylori* status in adult males. J Clin Endocrinol Metab.

[66] Gunji T, Matsuhashi N, Sato H, Fujibayashi K, Okumura M, Sasabe N, Urabe A (2008). *Helicobacter pylori* infection is significantly associated with metabolic syndrome in the Japanese population. Am J Gastroenterol.

[67] Isomoto H, Ueno H, Nishi Y, Wen CY, Nakazato M, Kohno S (2005). Impact of *Helicobacter pylori* infection on ghrelin and various neuroendocrine hormones in plasma. World J Gastroenterol.

[68] Nwokolo CU, Freshwater DA, O’Hare P, Randeva HS (2003). Plasma ghrelin following cure of *Helicobacter pylori*. Gut.

[69] Francois F, Roper J, Joseph N, Pei Z, Chhada A, Shak JR, de Perez AZ, Perez-Perez GI, Blaser MJ (2011). The effect of *H. pylori* eradication on meal-associated changes in plasma ghrelin and leptin. BMC Gastroenterol.

[70] Tschöp M, Smiley DL, Heiman ML (2000). Ghrelin induces adiposity in rodents. Nature.

[71] Halaas JL, Gajiwala KS, Maffei M, Cohen SL, Chait BT, Rabinowitz D, Lallone RL, Burley SK, Friedman JM (1995). Weight-reducing effects of the plasma protein encoded by the obese gene. Science.

[72] Ioannou GN, Weiss NS, Kearney DJ (2005). Is *Helicobacter pylori* seropositivity related to body mass index in the United States?. Aliment Pharmacol Ther.

[73] Cho I, Blaser MJ, François F, Mathew JP, Ye XY, Goldberg JD, Bini EJ (2005). *Helicobacter pylori* and overweight status in the United States: data from the third National Health and nutrition examination survey. Am J Epidemiol.

[74] Wu MS, Lee WJ, Wang HH, Huang SP, Lin JT (2005). A case-control study of association of *Helicobacter pylori* infection with morbid obesity in Taiwan. Arch Intern Med.

